# Linking Α to Ω: diverse and dynamic RNA-based mechanisms to regulate gene expression by 5′-to-3′ communication

**DOI:** 10.12688/f1000research.7913.1

**Published:** 2016-08-19

**Authors:** Megan E. Filbin, Jeffrey S. Kieft

**Affiliations:** 1 Department of Chemistry, Metropolitan State University of Denver, Denver, Colorado, 80217, USA; 2 Department of Biochemistry and Molecular Genetics, University of Colorado Denver School of Medicine, Aurora, Colorado, 80045, USA

**Keywords:** messenger RNA, polyadenylate tail, 7-methylguanosine, mRNA circularization

## Abstract

Communication between the 5′ and 3′ ends of a eukaryotic messenger RNA (mRNA) or viral genomic RNA is a ubiquitous and important strategy used to regulate gene expression. Although the canonical interaction between initiation factor proteins at the 5′ end of an mRNA and proteins bound to the polyadenylate tail at the 3′ end is well known, in fact there are many other strategies used in diverse ways. These strategies can involve “non-canonical” proteins, RNA structures, and direct RNA-RNA base-pairing between distal elements to achieve 5′-to-3′ communication. Likewise, the communication induced by these interactions influences a variety of processes linked to the use and fate of the RNA that contains them. Recent studies are revealing how dynamic these interactions are, possibly changing in response to cellular conditions or to link various phases of the mRNA’s life, from translation to decay. Thus, 5′-to-3′ communication is about more than just making a closed circle; the RNA elements and associated proteins are key players in controlling gene expression at the post-transcriptional level.

## Introduction

**Figure 1.**
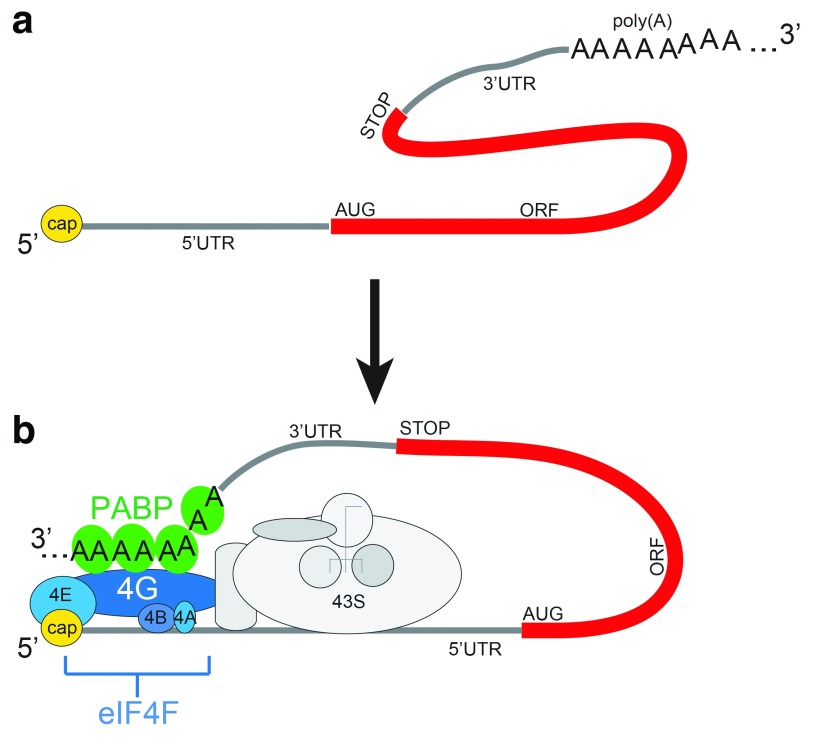
Canonical 5′-to-3′ communication in eukaryotic messenger RNAs (mRNAs). ( **a**) Diagram of a mature eukaryotic mRNA. The 5′ modified nucleotide cap is shown in yellow, the open reading frame (ORF) in red, and the start codon AUG, translation termination codon (STOP), poly(A) tail, and untranslated regions (UTRs) are labeled. ( **b**) Diagram of 5′-to-3′ communication in the context of translation initiation. Poly(A)-binding protein (PABP) is shown in green, the eukaryotic initiation factor (eIF) 4F complex is in shades of blue, and its components are labeled. The 43S complex is depicted in shades of gray. poly(A), polyadenylate.

Messenger RNAs (mRNAs) provide the template for synthesis of proteins; by definition, they contain an open reading frame (ORF) that encodes an amino acid sequence. However, encoding a protein is not enough: the use and fate of specific eukaryotic mRNAs must be controlled within the overall strategy used by cells to regulate gene expression. Much of the regulatory power is conferred by essential *cis*-acting sequences and structures in the mRNA’s untranslated regions (UTRs), which reside both 5′ (upstream) and 3′ (downstream) of the ORF. Also important for regulation, the vast majority of mature eukaryotic mRNAs have a modified nucleotide (generally a 7-methylguanosine, or m7G) on their 5′ terminus and a polyadenylate (poly[A]) tail on their 3′ end ( Figure 1a). These features control the fate of the mRNA, in part through long-range communication between the 5′ and 3′ ends. Often described as “mRNA circularization”, this phenomenon is central in controlling a number of post-transcriptional events. In addition to cellular mRNAs, many viral genomic RNAs use communication between their 5′ and 3′ ends, illustrating how useful, important, and ubiquitous this strategy is.

In this review, we discuss various strategies used by mRNAs and viral RNAs to enable communication between their 5′ and 3′ ends. We present these interactions in the context of the ways that they direct RNA function, including driving efficient translation, regulating mRNA stability and turnover, and promoting viral replication. We do not attempt a comprehensive assessment of all related literature, but rather present some illustrative examples to show how evolution has crafted a variety of ways to confer 5′-to-3′ communication, how useful this strategy is, and the dynamic nature of these interactions. We also highlight a few emerging ideas and areas that remain mysterious, encouraging further investigation.

## 5′-to-3′ communication used in translational control

### Overview of eukaryotic translation initiation: canonical and non-canonical

Probably the most well-known use of 5′-to-3′ communication is to promote or regulate translation of eukaryotic mRNAs. Although some have urged caution in broadly accepting that the 3′ end of an mRNA affects initiation from the 5′ end ^1^, continued experimentation supports the existence of this communication. To explain how 5′-to-3′ communication can occur, we first outline the canonical initiation mechanism. The eukaryotic initiation factor (eIF) 4F complex, which contains the cap-binding protein eIF4E and scaffold protein eIF4G, binds at the 5′ end ^2– 4^. Bound eIF4F recruits the 43S complex, which contains the 40S ribosomal subunit ^3^. This complex then scans the 5′ UTR to find the appropriate start codon ^4^, where GTP hydrolysis, release of eIFs, and 60S subunit joining create a translationally competent 80S ribosome ^5– 7^ (reviewed in 8– 10). As an alternative to this canonical cap- and scanning-dependent pathway, some mRNAs and viral RNAs contain an internal ribosome entry site (IRES) that directs translation initiation using a 5′ end-independent mechanism. IRES RNAs often use a subset of the canonical eIFs and RNA structures to drive translation initiation (reviewed in 11, 12).

### 5′-to-3′ communication in translation using canonical translation factors

A ubiquitous and important form of mRNA 5′-to-3′ communication depends on the 5′ cap and poly(A) tail ( Figure 1). The presence of these two signals on opposite ends of an mRNA synergistically enhances the rate of translation of that mRNA ^13, 14^. The model is that eIF4F binds to the cap at the 5′ end while multiple poly(A)-binding proteins (PABPs) bind the poly(A) tail and eIF4G, physically linking the 5′ and 3′ ends ( Figure 1b) ^15, 16^. Functionally, the PABP-eIF4G interaction is associated with favorable subunit binding at the 5′ end ^17, 18^, and it increases the affinity of cap-binding protein eIF4E for the cap ^19^. The PABP-eIF4G interaction can be regulated, supporting the importance of the 5′-to-3′ communication. For example, PABP-interacting proteins 1 and 2 (Paip1 and 2) either up- or down-regulate translation of an mRNA message by modulating the 5′-to-3′ communication ^20– 22^.

The PABP-eIF4G interaction within a single mRNA is the “closed loop model” ^23^, and it is proposed that the physical proximity between the 5′ and 3′ ends favors the transfer of terminating ribosomes from the 3′ end of the ORF to reinitiate at the 5′ end of the mRNA ^14, 15, 24^. One piece of evidence in support of this model comes from the observation that ribosomes within mature polysomes have a rate of exchange with surrounding free ribosomes that is slow enough to suggest reinitiation on an mRNA ^25^. In addition, the 5′-to-3′ interaction may serve as a quality-control mechanism to favor translation of full-length, mature mRNAs ^26^.

The canonical PABP-eIF4G circularization strategy appears to be a generally important mechanism, but care must be taken not to oversimplify its details or nuances. For example, the synergistic increase in translation efficiency conferred by the cap and poly(A) tail is not strictly contingent upon circularization. Specifically, addition of a poly(A) RNA to translationally competent extract *in trans* stimulates translation of a capped and non-polyadenylated mRNA to a similar degree as when the poly(A) tail is present *in cis*; this may be due to increased affinity of eIF4E for the cap ^27^. Also, in a series of recent publications, it has been observed that mRNAs go through changes in their global architectures over time and as the number of loaded ribosomes increases—first forming circles, then “double-row” structures, then complex “helical” structures where the ends may no longer interact—and there is evidence of similarly dynamic mRNA “closed-loops” in living cells ^25, 28– 30^. Even more interesting, when an mRNA is altered to prevent the PABP-eIF4G interaction, only initial rounds of translation initiation were slowed; the formation of higher-order polysome structures eventually occurred ^25, 31^. This suggests that the PABP-eIF4G interaction might be important for “kick-starting” the initial rounds of translation but is less important during steady-state translation on large polysomes, where other forces dictate higher-order architecture. This in turn suggests a dynamic model for 5′-to-3′ communication linked to the maturation of polysomes and perhaps other yet-to-be-determined signals. Clearly, there is much more to be learned even in regard to the canonical eIF4G-PABP interaction, especially surrounding events in live cells and how this relates to other events in the mRNA life, such as decay.

### Non-canonical 5′-to-3′ communication strategies using proteins

**Figure 2.**
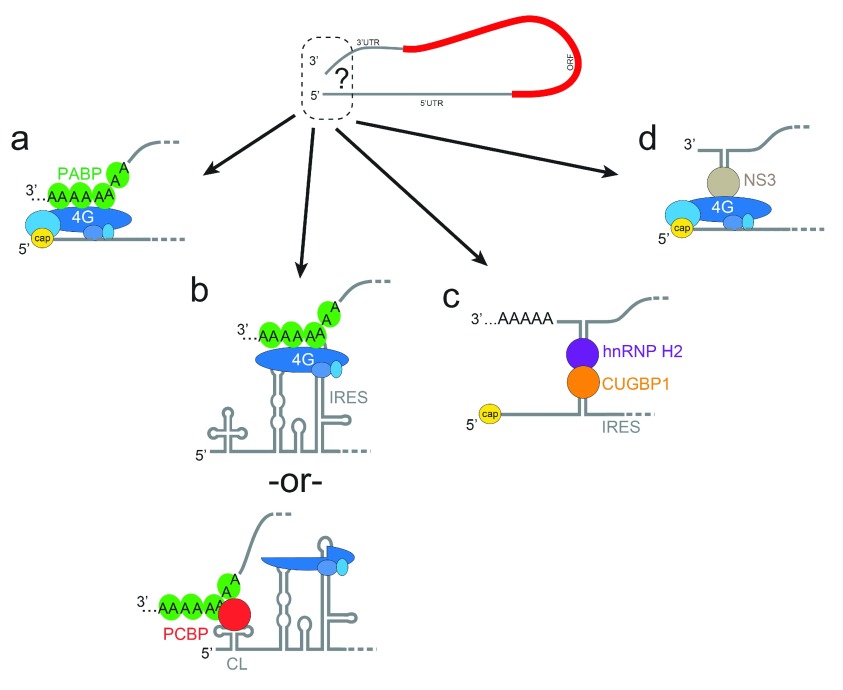
Diverse 5′-to-3′ communication strategies. ( **a**) The canonical eIF4G-PABP interaction, shown as in Figure 1b. ( **b**) Two potential strategies used by poliovirus. ( **c**) Strategy used by the serine hydroxymethyltransferase 1 IRES-containing mRNA. ( **d**) Strategy used by rotavirus. CL, cloverleaf; CUGBP1, CUG-binding protein 1; hnRNP H2, heterogeneous nuclear ribonucleoprotein H2; IRES, internal ribosome entry site; NS3, non-structural protein 3; ORF, open reading frame; PABP, polyadenylate-binding protein; PCBP, poly(rC)-binding protein.

Exploration of 5′-to-3′ communication starts with the canonical PABP-eIF4G interaction ( Figure 2a) but does not end there (pun intended). Other strategies include those used by IRES RNA-containing transcripts of viral origin that operate without a cap. For example, the viral RNA of poliovirus (and related viruses) is not capped, but translation of the viral genome is still enhanced by a 3′ poly(A) tail ^32^. The poliovirus IRES RNA directly binds eIF4G, which could interact with PABP bound to the poly(A) tail ( Figure 2b) ^33– 36^. However, during infection, a viral protease cleaves eIF4G and while a fragment of the factor binds the IRES, it cannot bind PABP. Thus, an alternative mechanism includes 5′-to-3′ communication between poly(rC)-binding protein (PCBP) bound to the 5′ cloverleaf structure, upstream of the IRES, and PABP bound to the 3′ poly(A) tail ( Figure 2b) ^37– 39^. In addition, it has been shown that sequences upstream of the poly(A) tail, within the 3′ UTR, can enhance translation in the absence of the poly(A) tail, perhaps by using other proteins ^40^. Thus, even within this one IRES-containing virus, there may be multiple ways that 5′-to-3′ end communication can be achieved. In addition, viral RNAs with a 5′ viral protein of the genome (VPg) in place of a 5′ cap structure, such as members of the *Calciviridae* family, might use VPg recruitment of the eIF4F complex (in particular, eIF4G, along with 4A and PABP) to mediate end-to-end communication ^41^. These different strategies may be used during different stages of viral infection, suggesting temporally dynamic interactions that respond to changing cellular conditions and help coordinate different viral processes, such as replication (discussed in more depth later in this review).

Viral IRESs are not the only places where alternative methods of 5′-to-3′ communication are found, as cellular mRNAs with IRESs also exhibit different strategies. An interesting example is found in the cellular c-myc mRNA, which contains an IRES that binds initiation factors, including eIF4G, and translation is enhanced by the poly(A) tail but *without* needing PABP ^42^. In this case (and many others), the precise mechanism of 5′-to-3′ communication is mysterious. Another intriguing example is the cellular mRNA for serine hydroxymethyltransferase 1, which does not require eIF4G or PABP. Rather, proposed structures in the 5′ and 3′ UTRs bind to heterogeneous nuclear ribonucleoprotein H2 (hnRNP H2) and CUG-binding protein 1 (CUGBP1), respectively, which mediate IRES-driven translation initiation via direct protein-protein contact during states of decreased cap-driven translation initiation ( Figure 2c) ^43^.

Although IRESs are logical places to use alternative modes of 5′-to-3′ communication, non-canonical mechanisms are also found in viral and cellular transcripts that do not contain IRESs. Rotaviruses are capped but do not have a poly(A) tail; rather, viral non-structural protein 3 (NSP3) binds near the 3′ end and interacts with eIF4G ( Figure 2d) ^43– 46^. Whereas earlier studies concluded that NSP3 was not involved in translation based on a partial knockdown ^47^, another interpretation of these data is that NSP3 is a potent translation enhancer, such that a 10-fold decrease in the amount of NSP3 levels still supports viral protein synthesis ^48^. Likewise, the cellular mRNAs that encode for histones have a highly conserved stem-loop structure at their 3′ ends rather than a poly(A) tail. This structure binds stem-loop-binding protein (also called hairpin-binding protein), which facilities interactions between initiation factors at the 5′ end ^49^. Clearly, the advantageous effect of 5′-to-3′ communication can be achieved with a variety of combinations of mRNA intrinsic elements (5′ cap, poly(A), and RNA structure), used with canonical factors (eIF4G and PABP), or several “non-canonical” proteins, or both. These mRNAs and viral RNAs illustrate how, despite the existence of the general eIF4G-PABP strategies, there are idiosyncratic methods of achieving 5′-to-3′ communication that add layers of regulatory complexity, which may be linked to cell type, cell conditions, and so on. No doubt there are many more strategies to be discovered and understood.

### Binding initiation factors at one end for use at the other: RNA-RNA communication and other strategies

**Figure 3.**
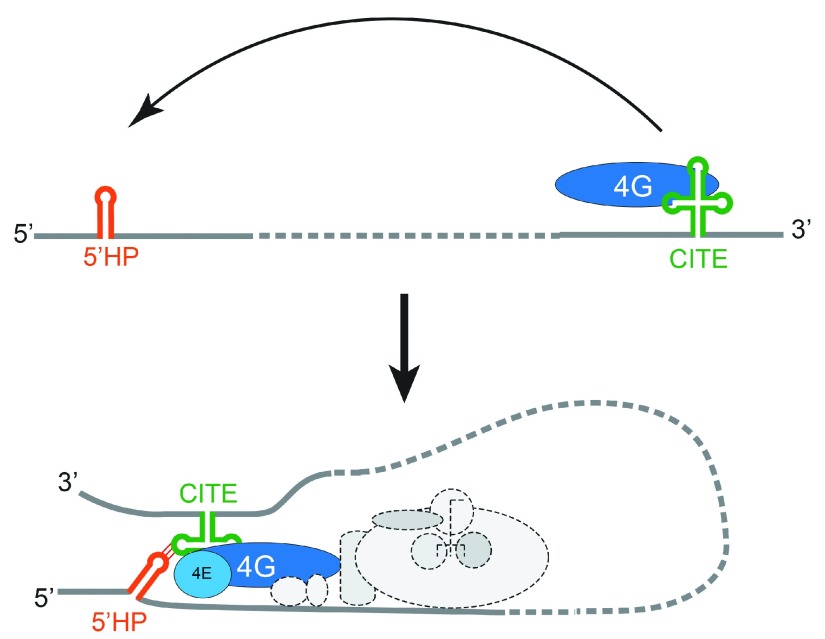
A strategy used by some CITE RNAs. (Top) The CITE element (green) is located in the 3′ untranslated region where it can bind a eukaryotic initiation factor (eIF) (in this case, eIF4G associated with eIF4E, both in blue). (Bottom) Base-pairing between a hairpin (HP) in the CITE and a HP near the 5′ end (orange) results in delivery of the factor to the 5′ end, where it can serve in translation initiation along with the 43S complex (shown in dashed gray). Other CITEs use diverse strategies. CITE, cap-independent translation enhancer.

The above examples illustrate how 5′ and 3′ ends communicate through various combinations of RNA-encoded signals and proteins. Interesting variations on this theme are RNA signals near the 3′ end of an RNA that bind translation-essential proteins to be used at the 5′ end ( Figure 3). Examples of this phenomena are found in plant-infecting viruses, whose genomic RNAs are often not capped or poly(A)-tailed. Rather than evolving an IRES, these viruses have structured RNA elements in their 3′ UTRs called cap-independent translation enhancers (CITEs) (reviewed in 50, 51). CITEs are distinct from IRESs in that CITEs themselves do not bind ribosomes in the proper location or context for translation initiation. The structures of CITEs and the mechanisms by which they operate are diverse and fall into several classes. This has been well reviewed recently ^51^; therefore, we present just a few examples.

CITEs generally eschew the use of proteins to achieve long-range communication, relying instead on direct RNA-RNA base-pairing between distal sequences. This streamlined approach often consists of a hairpin structure in the 3′ UTR that is complementary to four to eight bases in another short hairpin structure in the 5′ UTR (reviewed in 52). One of the best-characterized examples is from the non-capped and non-polyadenylated *Barley yellow dwarf virus* (BYDV), whose 3′ UTR contains a proposed cruciform-like secondary structure referred to as a BYDV-like element (BTE). One stem-loop of the BTE can base-pair to five nucleotides in a short 5′ UTR hairpin, creating a “kissing” interaction ^53^. Because the BTE binds to eIF4G directly, this provides a way to deliver eIF4G to the 5′ UTR ^54, 55^ and also possibly binds directly to the 18S rRNA through base-pairing to recruit the ribosome to the 5′ end ( Figure 3) ^56^. In fact, direct RNA-ribosome interaction is an emerging mechanism in viral and cellular mRNA translation ^57^. Another class of CITEs are *Panicum mosaic virus*-like translational enhancers (PTEs), which functionally replace a cap structure by directly binding eIF4E ^58– 60^. Although the high-resolution structure of a PTE-eIF4E complex has not been solved, it has been proposed that these RNAs form a compact fold in which a conserved guanine base is extruded from the structure to be recognized by the initiation factor ^61^. Consistent with the trend of many 3′ CITEs, there are sequences in the PTE that are complementary to sequences in the 5′ end, and these are likely part of the mechanism for bringing eIF4E to the 5′ end to be used in translation initiation.

The CITEs mentioned above have a fairly well-established strategy to link their 5′ and 3′ ends, but the mechanism for other CITEs is less straightforward. For example, the T-shaped structure (TSS) CITEs found in the genome of several viruses, including *Turnip crinkle virus*, are proposed to fold into a three-dimensional structure that resembles a tRNA ^62, 63^ which binds to 80S ribosomes and 60S subunits ^64^. Unlike the case in many other CITEs, there is no clear sequence complementarity between the TSS and sequence in the 5′ end; the current model is that 5′-to-3′ communication occurs through an unusual ribosome subunit-subunit interaction of the 3′ bound 60S subunit with a 40S subunit bound to the 5′ end ^65^. Clearly, the details of these interactions deserve continued exploration.

## End-to-end communication and messenger RNA turnover

Thus far, we have focused on the use of 5′-to-3′ communication in translation, but in fact the m7G cap structure and poly(A) tail serve more than one purpose: recruitment of translation machinery for protein synthesis and also protection against degradation of the RNA by exonucleases (reviewed in 66, 67). Consistent with this, communication between cap-bound and poly(A)-bound proteins can regulate RNA turnover. In the dominant pathway for mRNA decay, an mRNA that is destined for decay first has its poly(A) tail progressively shortened by deadenylating enzymes (deadenylation-dependent decay) ^68^. This shortening is accompanied by the loss of bound PABP, disrupting the 5′-to-3′ communication, likely loss of eIF4E affinity for the cap, enzymatic decapping, and degradation by the 5′-to-3′ exonuclease Xrn1 ^69, 70^. In a less used deadenylation-independent decay pathway, a stem-loop structure in the 3′ UTR of the mRNA binds a factor that enhances decapping (Edc3) at the 5′ end (and subsequent degradation) ^68^. The fact that translation and mRNA decay are both influenced by 5′-to-3′ communication connects and coordinates these processes within an overall regulatory strategy.

## End-to-end communication promotes viral replication

Unlike mRNAs, the genomic RNAs of positive-sense single-stranded viruses must be replicated to make a negative-sense RNA intermediate that serves as a template for many copies of the positive-sense RNA. Communication between the 5′ and 3′ ends is essential for this, and several strategies exist to achieve it. Again with poliovirus as an example, the RNA-dependent RNA polymerase (RNAP) must initiate minus-strand synthesis from the poly(A) tail. To do this, the cloverleaf RNA structure within the 5′ end of the viral RNA binds to PCBP and also to RNAP; both interact with poly(A)-binding protein 1, which binds the poly(A) tail ^71, 72^. These interactions support a model in which the protein-protein bridge helps specifically deliver RNAP recruited via unique structures in the 5′ end, to the 3′ end. This helps to distinguish poliovirus RNA from other poly(A) RNAs and ensures that only full-length poliovirus RNAs are replicated ^72^. A different mechanism with a similar outcome is found in Dengue virus (a flavivirus). In Dengue, direct base-pairing that forms between sequences in the 5′ and 3′ UTRs of the viral RNA do not appear to have a role in directing translation but are important for viral replication ^73, 74^. Specifically, a 5′ stem-loop functions as a promotor to bind the RNAP and the base-pairing then is thought to deliver the RNAP to the 3′ UTR to commence minus-strand synthesis ^75, 76^.

A general theme in many viral RNAs is that 5′-to-3′ communication plays a role in both translation and replication. Dynamic changes of the interactions between the 5′ and 3′ ends (either different RNA-RNA interactions or differentially bound bridging protein interactions) have been proposed as mechanisms to organize these processes ^77, 78^. This is important because the translation and replication machinery would clash should they both simultaneously use the same copy of viral RNA. This again underscores a common theme: that 5′ and 3′ communication strategies are dynamic and diverse players in regulating the use and fate of the associated RNA.

## Summary and final thoughts

In the preceding text, we have illustrated the diverse interaction strategies in which eukaryotic cellular mRNAs and some viral RNAs can promote communication between the 5′ and 3′ ends and how this relates to a number of different processes. Although there are common mechanisms by which communication is achieved, there are no “hard and fast” rules; examples of RNA-RNA, RNA-protein, and protein-protein interactions abound, and these examples use canonical factors as well as more specialized proteins. In some cases, the strategy appears to be idiosyncratic to a particular mRNA or virus, consistent with hundreds of millions of years of evolutionary tinkering and fine-tuning. Although many strategies have been described, it is clear that there are many others remaining to be discovered. Very recently, Weingarten-Gabbey *et al*. used a high-throughput approach to identify thousands of non-canoncial translation initiation signals in both viral and human RNAs located throughout the RNA; although interactions between these and the 5′ end have not been shown, these discoveries suggest that many more examples of long-range communication are still to be discovered ^57^. Also, there is much to be learned about how these interactions may be affected by changing cellular conditions, concentration of factors or RNA, cell type, time of viral infection, and so on. Even in the case of the canonical PABP-eIF4G interaction, quantitative assessment of the affinities, conformational changes and binding dynamics, and how these relate to the maturation of polysomes and transitions between different phases of the mRNA’s life cycle remain areas ripe for more exploration. Overall, there is much to learn about roles of higher-order architecture and the dynamics of these architectures. Happily, this suggests that many more exciting discoveries are on the horizon.
